# Impact of Surface Treatments on the Transport Properties of Germanium 2DHGs

**DOI:** 10.1021/acsaelm.5c01069

**Published:** 2025-09-25

**Authors:** Nikunj Sangwan, Eric Jutzi, Christian Olsen, Sarah Vogel, Arianna Nigro, Ilaria Zardo, Andrea Hofmann

**Affiliations:** † Department of Physics, 27209 University of Basel, Klingelbergstrasse 82, 4056 Basel, Switzerland; ‡ Swiss Nanoscience Institute, Klingelbergstrasse 82, 4056 Basel, Switzerland

**Keywords:** planar germanium, fabrication, surface cleaning, charge traps, Hall bar, cryogenic transport measurement

## Abstract

Holes in planar germanium (Ge) heterostructures show promise for quantum applications, particularly in superconducting and spin qubits, due to strong spin–orbit interaction, low effective mass, and the absence of valley degeneracies. However, charge traps cause issues such as gate hysteresis and charge noise. This study examines the effect of surface treatments on the accumulation behavior and transport properties of Ge-based two-dimensional hole gases (2DHGs). Oxygen plasma treatment reduces conduction in a setting without applied top gate voltage, improves the mobility, and lowers the percolation density, while hydrofluoric acid (HF) etching provides no benefit. The results suggest that interface traps from the partially oxidized silicon (Si) cap pin the Fermi level and that oxygen plasma reduces the trap density by fully oxidizing the Si cap. Therefore, optimizing surface treatments is crucial for minimizing the charge traps and thereby enhancing the device’s performance.

## Graphical abstract

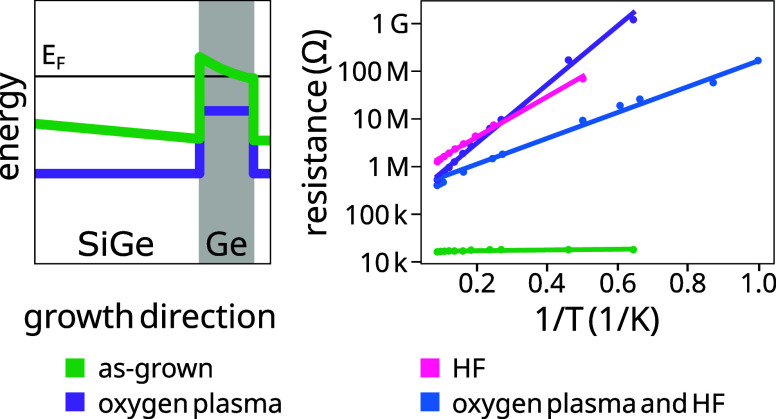

## Introduction

Holes in germanium (Ge) have garnered significant attention for quantum applications, particularly in superconducting^1–3^ and spin qubits.^4–7^ Their appeal comes from favorable material properties, including strong spin–orbit interaction (SOI),^8,9^ low effective mass, low hyperfine interaction, and the absence of valley degeneracies^10^ and piezoelectricity.^7^ Planar Ge heterostructures are known for their high hole mobility^11,12^ and low percolation density,^13,14^ and they promise scalable device architectures.^15^ The field has rapidly advanced from demonstrating single qubit operations^5,16^ and simple Josephson junctions^17–20^ to achieving coherent coupling of four qubits^6^ and realizing gatemon qubits.^2^ However, planar Ge heterostructures face challenges due to charge traps distributed throughout the stack. These traps lead to issues such as gate hysteresis,^21,22^ which complicates device operation, charge noise that limits coherence,^2,13,23–25^ and two-level fluctuators that induce loss channels for microwave resonators.^26^ Addressing these issues is crucial for improving device reproducibility and performance.

Charge traps are often introduced after the wafer is removed from the growth chamber and during the postgrowth fabrication processes, e.g. when clean surfaces are exposed to contaminants and Si and Ge form oxides. Cleaning steps, such as O_2_ plasma treatment or HF etching, have been shown to remove organic impurities, dangling bonds, and oxides, creating smoother and cleaner interfaces for Si and, to some extent, Ge surfaces.^27–29^ Additionally, the gate dielectric, typically aluminum oxide (Al_2_O_3_), can host impurities. The quality of the gate dielectric depends on the deposition and annealing conditions.^30–35^

## Experimental Section

In this study, we systematically investigate the impact of surface treatments, oxide (Al_2_O_3_) deposition, and oxide annealing conditions on the equilibrium energy levels and the transport behavior of the 2DHG in a Ge quantum well (QW). Even though all our heterostructures are undoped, we observe that some fabrication schemes induce a conducting channel at zero top gate voltage, while others do not. We propose a straightforward explanation based on the strong Fermi level pinning in Ge. This framework also explains the variations in gate efficiency, mobility, and percolation density observed in devices subjected to different surface treatments.

A cross-sectional schematic of the reverse graded Ge/SiGe heterostructure used in this study is shown in Figure 1a. The heterostructure was grown via chemical vapor deposition (CVD) using the reverse grading approach, in a commercial cold wall CVD PlasmaPro 100 Nanofab reactor equipped with a showerhead (Oxford Instruments, base pressure < 0.5 mTorr), as detailed in ref 14. A 500 nm thick Ge virtual substrate was deposited on a (100) Si substrate by a two-step temperature growth. This was followed by a reverse linearly graded Si_1–*x*_Ge_*x*_ alloy, with Ge content (*x*) varying from 1 to 0.8. A 14 nm thick Ge quantum well was deposited between two Si_0.2_Ge_0.8_ barriers, with thicknesses of 300 and 55 nm, respectively. The structure was capped with a 1.5 nm thick Si layer. Unless specified otherwise, an in situ oxidization of the cap was performed in the CVD reactor chamber by flowing O_2_ at 500 °C and a partial pressure of 1 Torr for 5 min. No plasma was used during this step. The resulting structure exhibited a threading dislocation density of (6.0 ± 0.8) × 10^8^ cm^–2^, measured at the top of the SiGe graded layer.^26^

**Figure 1:**
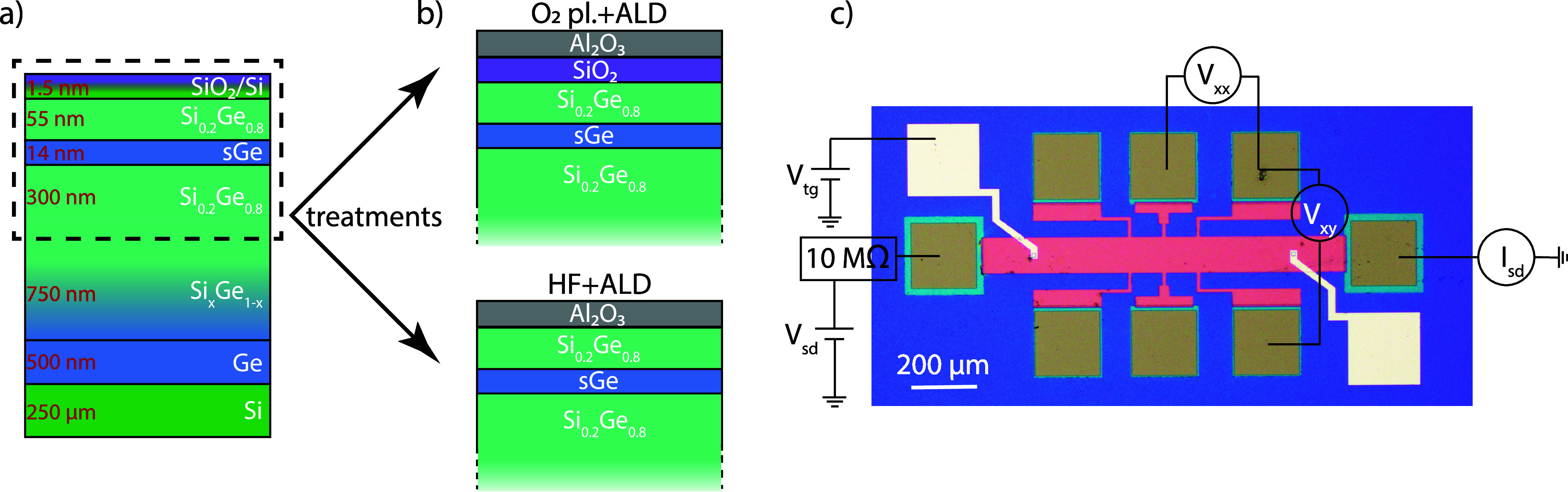
Device schematics: (a) Cross-section of the reverse graded Ge heterostructure with a strained Ge (sGe) QW, and (b) the effects of the surface treatments on the Si cap of the Ge heterostructure. (c) Measurement schematic of a single-gated layer Hall bar device.

We fabricated two types of devices, a set of simple devices with PtSiGe ohmic contacts and another set of devices with an additional Al_2_O_3_ gate-oxide and a top gate. The fabrication closely follows the recipe described in refs 14, 36 . Four different types of samples were fabricated, labeled “O_2_”, “HF”, “O_2_ + HF” or “as-grown”. The devices with the surface treatment “O_2_” were oxidized in an O_2_ plasma before any fabrication step. The devices with the surface treatment “HF” were dipped in a 2.3% HF solution after the deposition of ohmic contacts and directly before growing the aluminum oxide. The treatment “O_2_ + HF” involves the combination of both surface treatments mentioned above, i.e. “O_2_” followed by contact deposition and then “HF” before depositing the Al_2_O_3_. No treatment was performed for the “as-grown” devices. We use a layer of silicon nitride (SiN_*x*_) as a bond-pad protection for our gated devices. The SiN_*x*_ deposition at 300 °C also acts as a contact annealing step. For our ungated devices, where no SiN_*x*_ is deposited, the ohmic contacts were annealed in forming gas before the Al_2_O_3_ growth. Due to the strong Fermi level pinning of Ge, the ohmic contact resistance measured typically amounts to a few k Ω.^36^ A flow-chart outlining the processing steps for each surface treatment is provided in ref 36.

## Results and Discussion

### Ungated Devices

In the initial set of experiments, we study the simple devices without a top gate and verify whether charges are accumulated in the undoped QW for various device configurations as described in the following. We evaluate two surface treatments, labeled “as-grown” and “O_2_”, and study how the presence of Al_2_O_3_ and its growth temperature influence the properties of the device. For all samples, the contacts have been annealed prior to any measurement. For each configuration, we perform two-probe measurements at a temperature of *T* = 1.5 K of various contact pairs and plot the average resistance in Figure 2a. Additional measurements have been performed to verify that the measured conductance is due to holes in the QW rather than any conducting channel on the surface.^36^ The results in Figure 2a illustrate that the “O_2_” devices have a higher resistance (*R* ≥ 15 MΩ) than the “as-grown” devices (*R* ∼ 1 kΩ). In contrast, neither the presence of the Al_2_O_3_ nor its deposition temperature dramatically change the resistance of the sample. These results indicate that the mechanism responsible for the measured conduction do not originate in the deposited oxide but in surface-near layers in the wafer.

**Figure 2:**
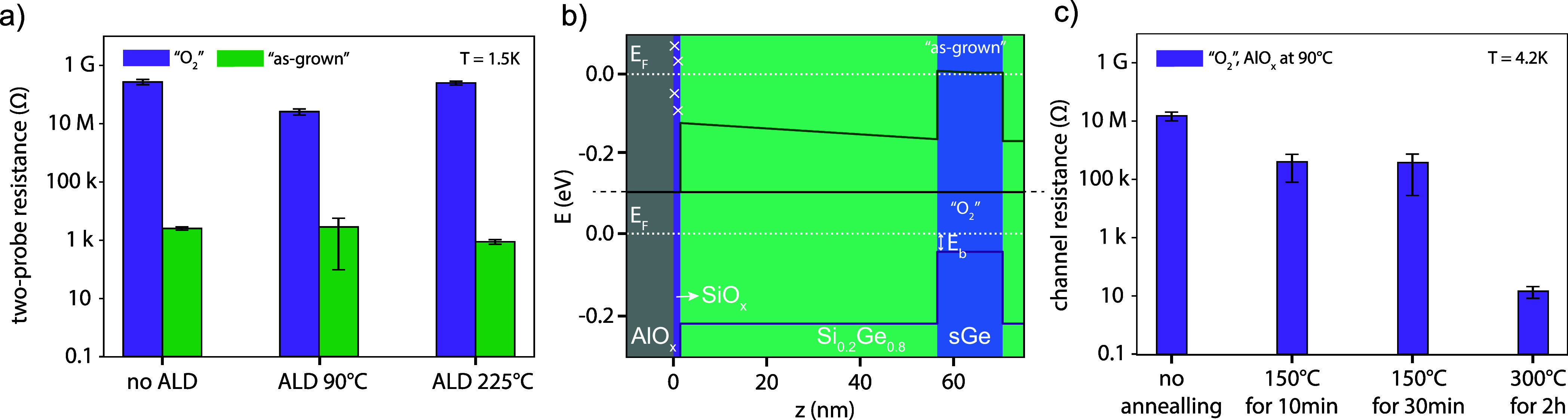
Ungated devices: (a) Two-probe resistance of ohmic contact pairs for different deposition temperatures and treatments “as-grown” and “O_2_”. The second and third pair of bars show the results with Al_2_O_3_ deposited at temperatures of 90 °C and 225 °C. (b) Representational valence band energy diagrams for two cases. Top: The valence is bent due to the Fermi level pinning to the charge traps. Bottom: Band diagram expected without trap states. (c) Channel resistance for different annealing conditions.

It is well-known that unpassivated Ge easily forms surface states, leading to a strong Fermi level pinning^37^ if enough charges are available. Typically, the charge neutrality level in Ge metal-oxide semiconductor (MOS) structures amounts to ∼0.1 eV above the valence band.^37^ The resulting band bending may be strong enough to move the valence band above the Fermi energy for our heterostructures, thereby inducing a 2DHG without a top gate. Two possible scenarios are shown in Figure[Fig fig2]b. The top part of the figure displays how the presence of the interface states together with the strong Fermi level pinning in Ge lead to a significant band bending such that the valence band rises above the Fermi level in the QW. On the other hand, the bottom part shows that the valence band remains below the Fermi level in the presence of only few impurities. Nevertheless, the energy difference can be small enough to allow for thermal excitation of charge carriers in the QW even at low temperatures. The conduction of the ungated QW may therefore be used as an estimate of the amount of charge traps close to the wafer surface. The large resistance measured in the “O_2_” devices indicates that the valence band remains below the Fermi level, while the low resistance in the “as-grown” devices hints to the accumulation of a 2DHG even in the absence of a top gate. The large amount of surface states in the latter case is likely caused by incomplete oxidation of the Si cap. Even though 1.5 nm of Si are expected to completely oxidize in air,^38,39^ our amorphous Si cap may remain partially unoxidized due to the growth conditions in which it has been deposited^40^ or due to uncertainties in its thickness. In contrast, the oxygen plasma helps to fully oxidize the Si cap and, it removes any kind of residual polymers, as supported by additional X-ray photoelectron spectroscopy (XPS) and atomic force microscopy (AFM) data.^36^ Both effects contribute to reducing the amount of impurities, thereby resulting in a smaller band bending.

Further, we studied the effect of annealing the Al_2_O_3_ on the amount of charge traps. For this study, we used a heterostructure without in-chamber oxidation, fabricated “O_2_” devices, annealed the contacts at 300 °C for 50 min and deposited Al_2_O_3_ at 90 °C. The oxide was then further annealed at different temperatures and for different durations. Figure 2c shows the average and standard deviation of the 2DHG channel resistance measured at *T* = 4.2 K. Due to the high channel resistance for the first three conditions, the data are obtained from two-probe measurements subtracting the line resistance as well as an estimated contact resistance of 0.7 kΩ per contact.^36^ For the data obtained after annealing for 2 h at 300 °C, the channel resistance is directly extracted from four-probe measurements. The channel resistance decreases with increasing annealing temperature and time, indicating a larger amount of charge traps. It is known that annealing Al_2_O_3_ on Si increases the amount of fixed charges and decreases the amount of interface charges.^41–44^ From our data, we conclude that the combined effect is an increased band bending that induces a 2DHG in the QW when the annealing temperature and time are high enough.

### Gated Devices

We now focus on the effects of different surface treatments on the transport properties such as the 2DHG density, mobility and percolation density. Hall bar devices as shown in Figure 1c with all the four mentioned surface treatments were fabricated. A mesa was etched for the “O_2_” and “as-grown” devices. Standard lock-in magnetotransport measurements were performed to extract the transport properties from the measured longitudinal and Hall resistances at temperatures *T* ≤ 4.2 K (for exact temperatures see ref 36), where the mobility versus density is independent of temperature.^36,45^

We first study the gate efficiency by quantifying how much the density changes in response to a given variation in top gate voltage. Figure[Fig fig3]a shows the density as a function of top gate voltage recorded in a regime where we have access to the full range of densities but the density still increases linearly with the gate voltage.^36^ From the slope, we extract a gate efficiency of 5.51 × 10^11^ cm^–2^/V for the “as-grown” sample and 6.53 × 10^11^ cm^–2^/V for the devices with surface treatment. As reported in refs 21, 46, 47 the gate capacitance is decreased by the presence of charge traps. The lower gate efficiency measured in the “as-grown” devices is, therefore, again a signature of the increased amount of charge traps compared to the devices with surface treatment. The traps are filled from QW states, thus screening the top gate voltage. In subsequent top gate sweeps, the same density then is obtained at more negative voltages. Eventually, the charge tunnelling and the subsequent screening of the gate voltage prohibit a further increase in 2DHG density. In our experience, a complete reset can only be achieved by a thermal cycle to room temperature.^22,36^

**Figure 3:**
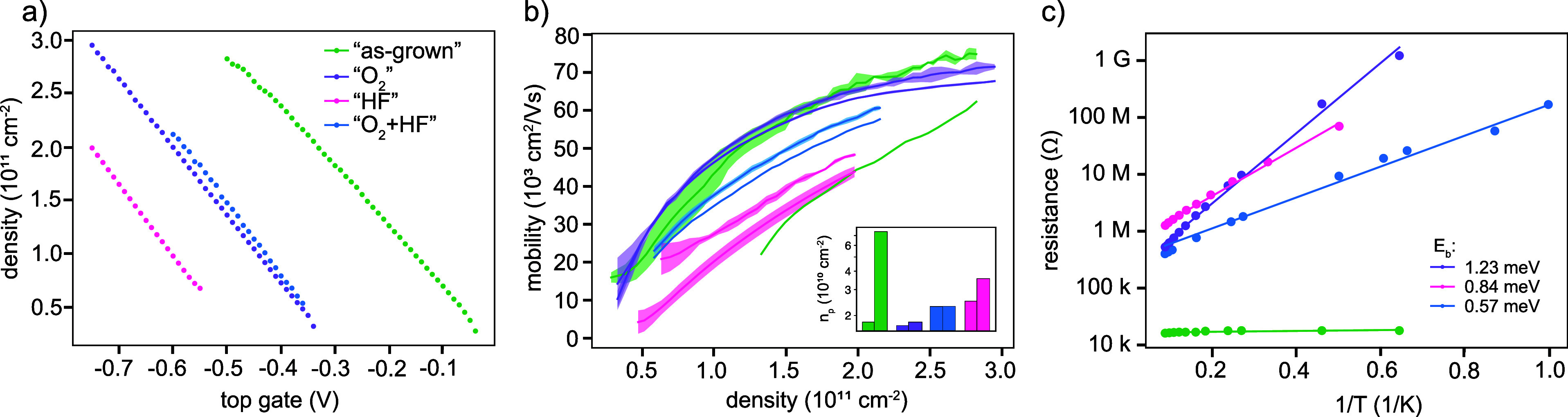
Hall bar devices: (a) Hall densities as a function of top gate for all treatments, (b) Hall mobilities as a function of Hall density and percolation density (*n*_p_), (inset) for all treatments. The two different data set per treatment show results from two different devices. The green data set with the lowest mobilities has no error bars, as for this device we only measured one Hall trace. (c) Two-probe resistance of ohmic contact pair as a function of temperature at zero top gate voltage for different surface treatments.

Next, we study how the surface treatment influences the mobility and the percolation density. In general, for our heterostructures, the mobility is limited by remote scatterers in the low-density regime, and by close-by scatterers in the high-density regime.^36,48^ For each surface treatment, the mobility has been measured in different top gate voltage configurations, corresponding to regions 1–3 in refs 21, 36 in at least two devices per treatment. In region 1, the density in the QW responds linearly to the applied gate voltage, before tunnelling-induced density shifts occur. In region 2 the density in the QW still increases but due to Fowler-Nordheim tunnelling the trap states at the surface of the heterostructure start to be populated. In region 3, the maximum density in the QW stops increasing and any additional charges populate interface trap states. The average mobility and standard deviation obtained from all the sweeps in the regimes 2–3 are plotted in Figure[Fig fig3]b, which show that “O_2_” consistently has the highest mobilities, followed by “O_2_ + HF” and “HF”. Meanwhile, the “as-grown” data show a larger variability within a device as well as in-between the two devices. We conclude that the “O_2_” devices have fewer charge traps than those treated with HF. Knowing that HF does not effectively passivate Ge,^27,29^ we suspect that additional traps are created when the Ge-rich heterostructure interface is exposed to air while moving it from the HF-solution to the oxide deposition chamber.^49^ The values extracted for the percolation density^36,50^ again corroborate our previous findings: “O_2_” devices have the smallest percolation density hinting at a small number of charge traps, while the HF treatment seems to induce more traps and hence a larger percolation density. The “as-grown” devices again show variable performance. This variability indicates that the potential landscape induced by the large number of traps may vary significantly in the heterostructure. Some regions might have a rather homogeneous potential landscape without deep traps, leading to mobilities and percolation densities comparable to the “O_2_” devices. In other regions, the presence of a larger amount and an inhomogeneous distribution of deep traps may cause lower mobilities and higher percolation densities.

As described in our model in Figure 2b and further detailed in simulations,^36^ the charge traps at the interfaces, together with the strong Fermi level pinning in Ge, lead to sizable band bending. We now quantify the amount of bending by estimating the energy difference *E*_b_ between the valence band and the Fermi level at zero applied top gate voltage (using the gated devices). Specifically, we measure the two-probe resistance as a function of temperature in the range from 1 to 15 K, and we find a thermally activated behavior. The resistances are plotted in Figure 3c together with a fit to ln­(*R*) = ln­(*R*_0_) + *E*_b_/*k*_B_ *T* where *k*_B_ is the Boltzmann constant and *R*_0_ and *E*_b_ are fitting parameters. From the measured activated behavior, we suspect that for low enough energies *E*_b_, thermal activation of charges can lead to the conduction in the 2DHG even in the absence of top gate voltages. In consistency with the previous data, the “as-grown” device has a low resistance that is independent of the temperature, indicating the accumulation of a 2DHG already at the lowest temperatures. Meanwhile, the other devices showed an activated behavior with *E*_b_ amounting to 1.23, 0.84, and 0.57 meV for the “O_2_”, “HF” and “O_2_ + HF” devices, respectively.

## Conclusions

In conclusion, our investigation of surface treatments and their effects on planar Ge heterostructures has provided valuable insights into the sources of charge traps and their impact on the device performance. We suspect that the Si cap is only partially oxidized and that this leads to a significant number of interface trap states, which degrade the electronic properties of the devices. Although HF cleaning is commonly used to remove oxides, it is ineffective in this context as it leaves partially unoxidized Si intact. Our study indicates that oxygen plasma treatment is more effective, as it fully oxidizes the Si cap, reducing the number of interface traps and improving the overall quality of the interface. However, when HF cleaning is combined with oxygen plasma treatment, the Ge-rich interface is exposed to air and contaminants, introducing new defects and undermining the benefits of the treatment.

The strong Fermi level pinning in Ge provides a reasonable explanation for our observation that devices with numerous defects exhibit finite conduction even without a top gate. Furthermore, the thermally activated behavior we observed indicates that oxygen plasma treatment results in the fewest defects, leading to the least band bending and reproducibly best device operation. These findings underscore the importance of selecting appropriate surface treatments to minimize charge traps and optimize the performance and reproducibility of Ge-based quantum devices.

## Supplementary Material



## Data Availability

The data that support the findings of this study are openly available in ZENODO at https://doi.org/10.5281/zenodo.17018065.

## Notes

Fabrication details, interface conduction tests, mobility vs density information, percolation density extraction, contact resistances, and additional XPS and AFM data (PDF)
