# Supplementary material for: Impact of Surface Treatments on the Transport Properties of Germanium 2DHGs
Source: ACS Appl Electron Mater. 2025 Sep 25;7(19):8844–9. doi: 10.1021/acsaelm.5c01069 (PMC12529946; doi:10.1021/acsaelm.5c01069)
Supplement: Supplementary file 1 [file el5c01069_si_001.pdf]

Supporting Information: Impact of surface treatments on the transport properties of germanium 2DHGs

Nikunj Sangwan,[†,‡] Eric Jutzi,[†,‡] Christian Olsen,[†] Sarah Vogel,[†] Arianna Nigro,[†] Ilaria Zardo,[†,¶] and Andrea Hofmann[*,†,¶]

†*Department of Physics, University of Basel, Klingelbergstrasse 82, 4056 Basel*

‡*contributed equally to this work*

¶*Swiss Nanoscience Institute, Klingelbergstrasse 82, 4056 Basel*

E-mail: andrea.hofmann@unibas.ch

Fabrication Details

Simple, ungated devices ("as-grown" and "O_2"):

1. Oxygen plasma only for "O_2" devices: The samples are exposed to 10 min of O_2 plasma at 60 W in an Oxford PlasmaPro 80 RIE at a O_2 flow of 20 sccm and a partial pressure of 60 mTorr.

2. Contact deposition: The SiO_x is removed in the exposed region by Ar milling. Electron beam evaporation of 20 nm of Pt follows in the same machine without breaking vacuum.

3. Contact annealing: The Pt is annealed for 50 min at 300°C in forming gas at a pressure of 21 mbar to diffuse the Pt to the quantum well (QW) and form ohmic contacts.

4. First measurements are performed (Main Figure 2(a), "no atomic layer depostion (ALD)")

5. Oxide deposition: 30 nm (Main Figure 2(a)) and 20 nm (Main Figure 2(c)) of aluminum oxide (Al_2O_3) is grown in an ALD process at either 90 °C or 225 °C.

6. Further annealing: Devices with oxide grown at 90 °C are further annealed in forming gas at a pressure of 21 mbar in conditions (temperature and time) as indicated (Main Figure 2(c)).

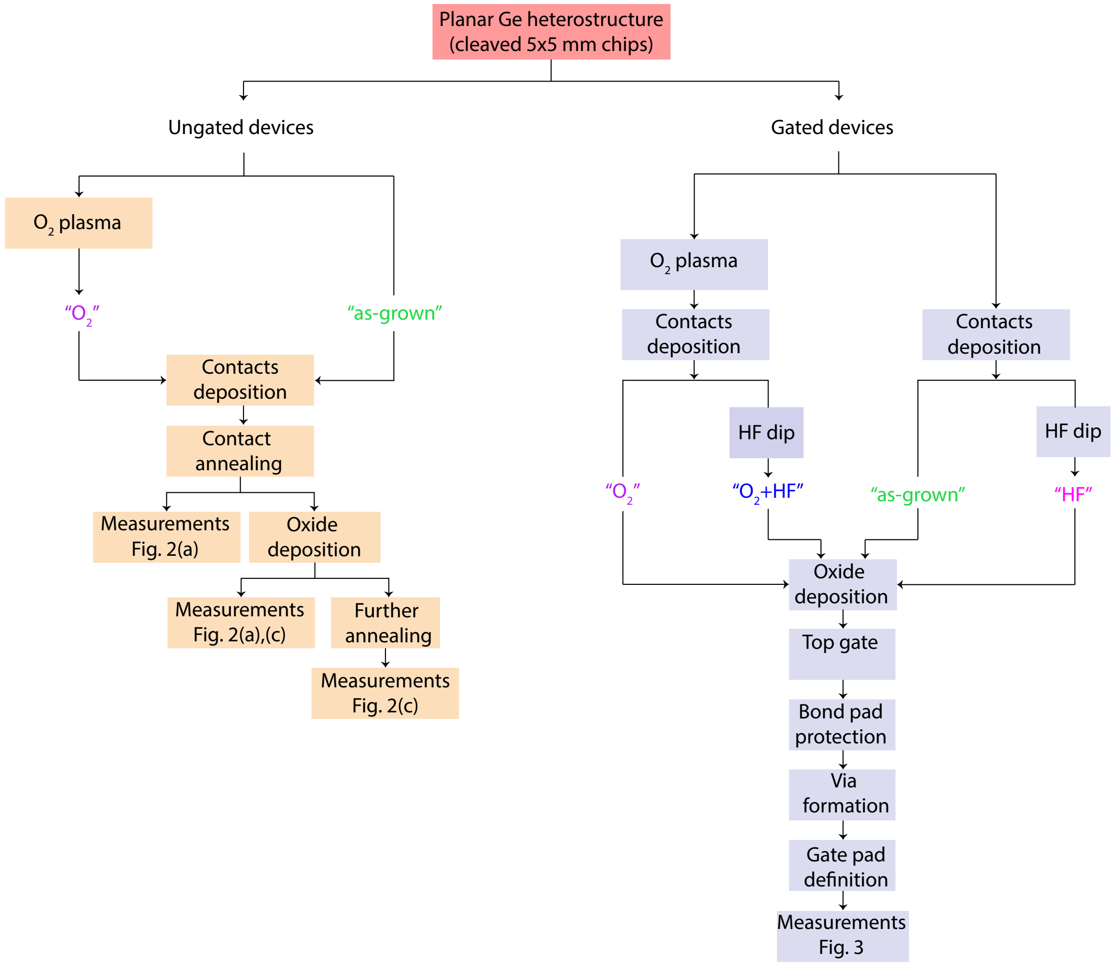

Fig. S1: Flow chart of the fabrication procedure

Gated devices:

1. Oxygen plasma only for "O_2" and "O_2+HF" devices: The samples are exposed to 10 min of O_2 plasma at 60 W in an Oxford PlasmaPro 80 RIE at a O_2 flow of 20 sccm and a partial pressure of 60 mTorr.

2. Contact deposition: Contact regions are defined using UV lithography. The SiO_x is removed in the exposed region by Ar milling. Electron beam evaporation of 20 nm of Pt follows in the same machine without breaking vacuum.

3. HF-dip only for "HF" and "O_2+HF" devices: The samples are dipped for 60 s in 2.3 % hydrofluoric acid (HF), rinsed for 10 s in deionized water and then submerged in isopropanol for transportation to the ALD chamber: they are blow dried immediately before loading them into the ALD chamber to minimize exposure of the surface to air. No protection layer was used because Pt is resistant to HF etching [1].

4. Oxide deposition: 30 nm of Al_2O_3 is grown in an ALD process at 225 °C.

5. Top gates: Top gate regions are defined by UV lithography. Ti/Au gates of thickness 5 nm/30 nm is deposited using electron beam evaporation

6. Bond pad protection: 216 nm of Si_3N_4 is grown at 300 °C. This process takes 45 min to 50 min with some variability due to manual unloading. This step also also serves as an annealing step for the ohmic contacts.

7. Via formation: The Si_3N_4 is removed above the ohmic contacts and on two small squares on the Ti/Au gate by reactive ion etching. The mask is defined by UV lithography.

8. Bond pad definition: Bond pad regions for the top gate are defined using UV lithography. Bond pads of 5 nm/250 nm Ti/Al are deposited using electron beam evaporation.

Contact Resistance

Using the gated devices, the two dimensional hole gas (2DHG) has been fully accumulated and the resistance of the channel has been extracted from a four-probe measurement. In the same configuration, the two-probe resistance has been measured. The line-resistance and the channel resistance have been subtracted, yielding an upper bound of the contact resistances. All measured contact resistances were below $7\,\mathrm{k\Omega}$ with an average of $3.6{\pm}1.9\,\mathrm{k\Omega}$.

For the "as-grown" devices, which are accumulated without gating, the contact resistance was also extracted for the ungated devices. The dedicated contact annealing leads to even lower contact resistances of $0.5\,\mathrm{k\Omega}$ to $0.7\,\mathrm{k\Omega}$ measured before any oxide deposition. No significant decrease was observed with oxide deposition at $90\,°\mathrm{C}$ or $225\,°\mathrm{C}$.

Interface conduction tests

Our conclusions of the results obtained with the simple devices relies on the assumption that the measured conduction occurs in the 2DHG rather than in any potentially conducting oxide interface. Therefore, we fabricated devices with contacts to the SiGe/SiO_x interface but not reaching the 2DHG. We found no conduction between any of these contacts. Additionally, on the conducting "as-grown" devices, we performed four-probe magnetotransport measurements and we clearly saw the oscillating behaviour resulting from the Shubnikov-de Haas effect of a high-mobility channel which was, however, not confined into a proper Hall bar.

Accumulation shift

A recent work [2] reported the observation of shifts in density accumulation with respect to the gate voltage which could only be reset by thermal cycling. We observe similar shifts, as shown in Figure S2. Each curve in Figure S2 shows the data where the top gate was swept from zero to a certain minimum value. The initial accumulation happens in the so-called

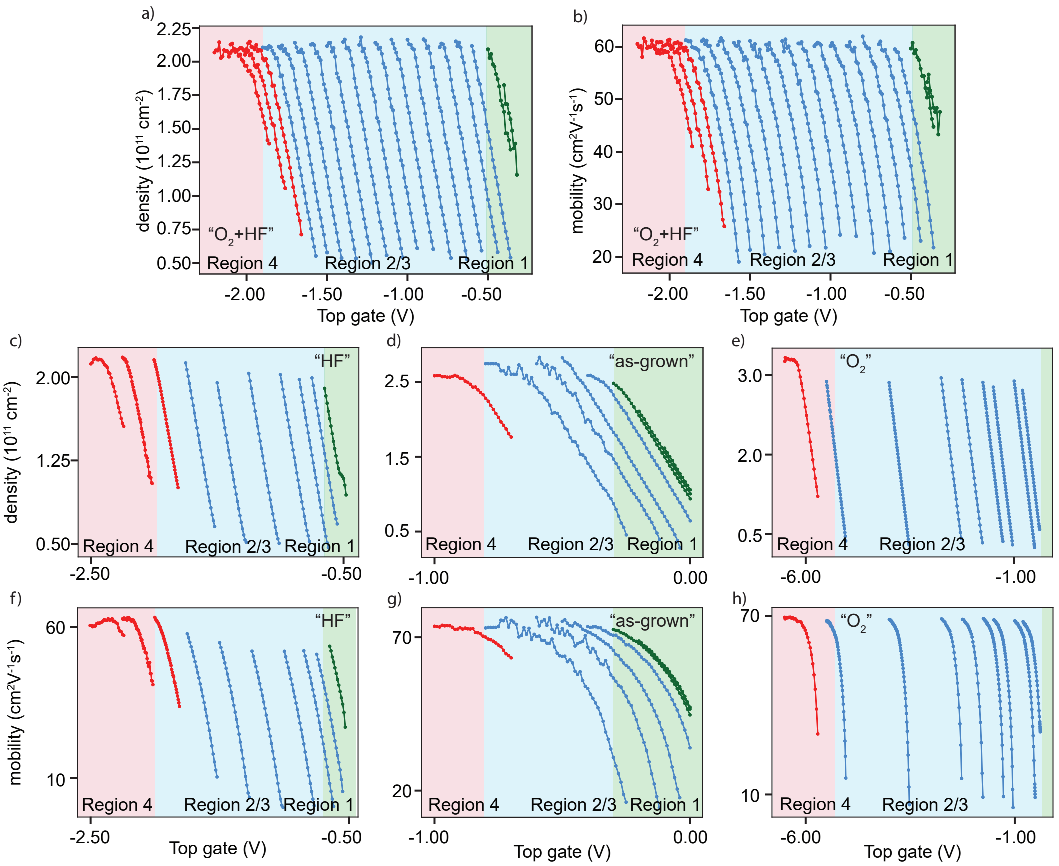

Fig. S2: Accumulation shift: Hall densities as a function of top gate voltage are plotted in (a) and (c)-(e) for the devices "O_2+HF", "HF", "as-grown", and "O_2", respectively. The gate voltage used for charge accumulation shifts to more negative values due to charges being trapped at surface-near layers. The corresponding Hall mobilities are shown in panels (b), (f)-(h).

region 1, c.f. [2]. Sweeping the top gate voltage to more negative values allows charges to tunnel into interface states. They screen the top gate potential and hence, the same density is reached at more negative values of the top gate voltage (region 2 and 3 in [2]). This tunnelling-induced shift continues until a triangular quantum well forms at the interface (region 4 in [2]). Filling these interface states creates a large disorder potential and poor low-density characteristics, prohibiting the extraction of density and mobility.

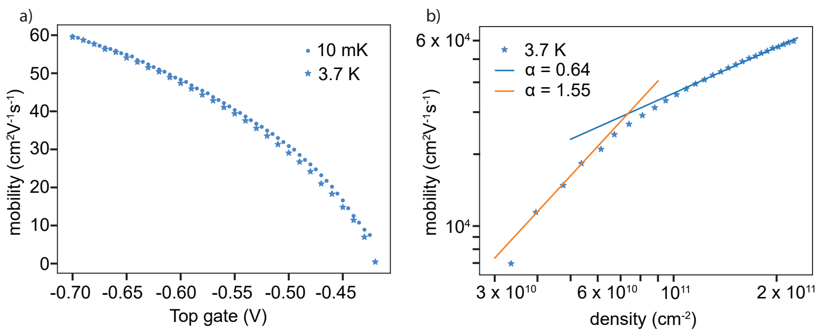

Fig. S3: (a) Mobility as a function of top gate voltage for the "O_2+HF" Hall bar. (b) Hall mobility as a function of Hall density for the same device with the fits following $\mu \propto n^{\alpha}$

Mobility vs temperature and different scattering regimes

The Hall measurements for Figure 3(a) are performed at a temperature of 1.5 K for the "as-grown", "O_2", "O_2+HF" devices, and at 4.2 K for the "HF" device. Fig. 3(b) contains data from two devices per surface treatment, with one of them measured at the respective temperature mentioned above and the other measured at $\sim$ 15 mK. Figure S3(a) shows the mobility of an "O_2+HF" device at two different measurement temperatures, namely 3.7 K and 10 mK. The independence of the mobility with respect to these low temperatures indicates that phonon-scattering is not the limiting scattering mechanism [3].

Figure S3(b) shows that at low density, the mobility is limited by scattering at interface states and traps nearby the QW. Meanwhile, at high density, it is limited by the background scatterers [4, 5].

Percolation density fits

The percolation density n_p can be estimated by fitting the conductivity in the low-density regime with the function $\sigma_{xx} \propto (n - n_p)^{\alpha}$ $\{\alpha \sim 1.31$ [6]$\}$, which is valid in the low-density regime. For fitting n_p, this low-density regime first has to be determined. We use the function mentioned above to fit our data with the following procedure: we first fit our data in two different ways, once by using two fit parameters (α and n_p) and once with only one fit

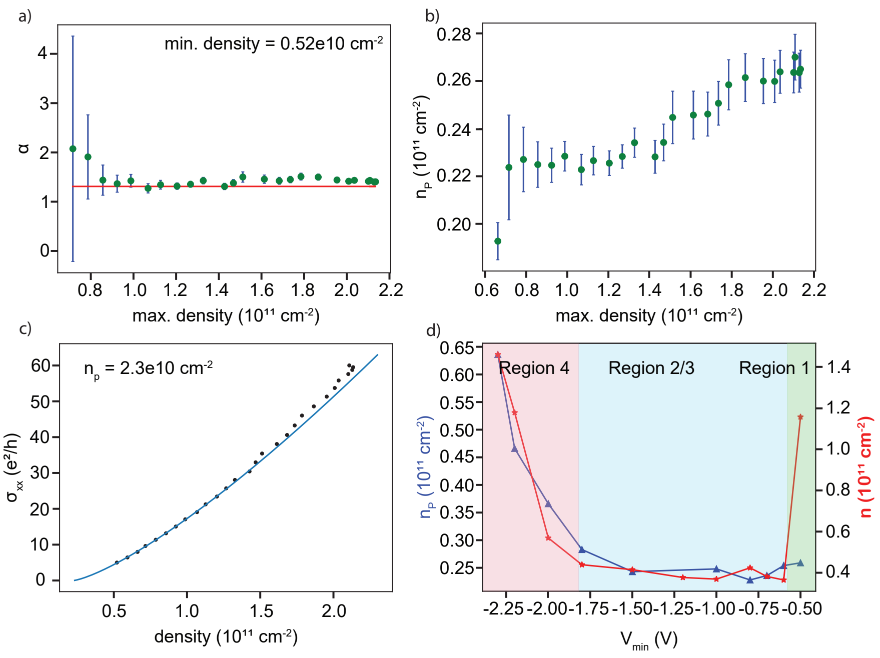

Fig. S4: Percolation density fits: Fit parameter (a) α, and (b) percolation density (n_p) from fitting $\sigma_{xx} \propto (n - n_p)^{\alpha}$ and $\sigma_{xx} \propto (n - n_p)^{1.31}$, respectively, as a function of maximum density used for the fit. (c) Example of fitting n_p using the accurate density range. (d) n_p and measured density at different shifted depletion points for an "O_2+HF" device.

parameter (n_p, while fixing $\alpha = 1.31$). Both these fits are performed with an increasing range of density used as input values. Examples are shown in Figure S4(a) and (b), respectively. Second, we select the density range such that $\alpha = 1.31$ and the error bars of the n_p fit with $\alpha = 1.31$ remain constant. We then use the selected regime to fit $\sigma_{xx} \propto (n - n_p)^{\alpha}$ with n_p as the only fit parameter and fixed $\alpha = 1.31$. Extracting percolation density for all the shifted accumulation curves suggests the same regions as mentioned in the section above.

Surface characterization by XPS and AFM

To study the effects of the surface treatments on the composition and roughness of the surface, XPS and AFM measurements were performed on the "as-grown" and "O_2" samples. The AFM measurements are performed on three different areas, each of size 10 µm^2. The average values of roughness measured on the large scan are within the precision provided by

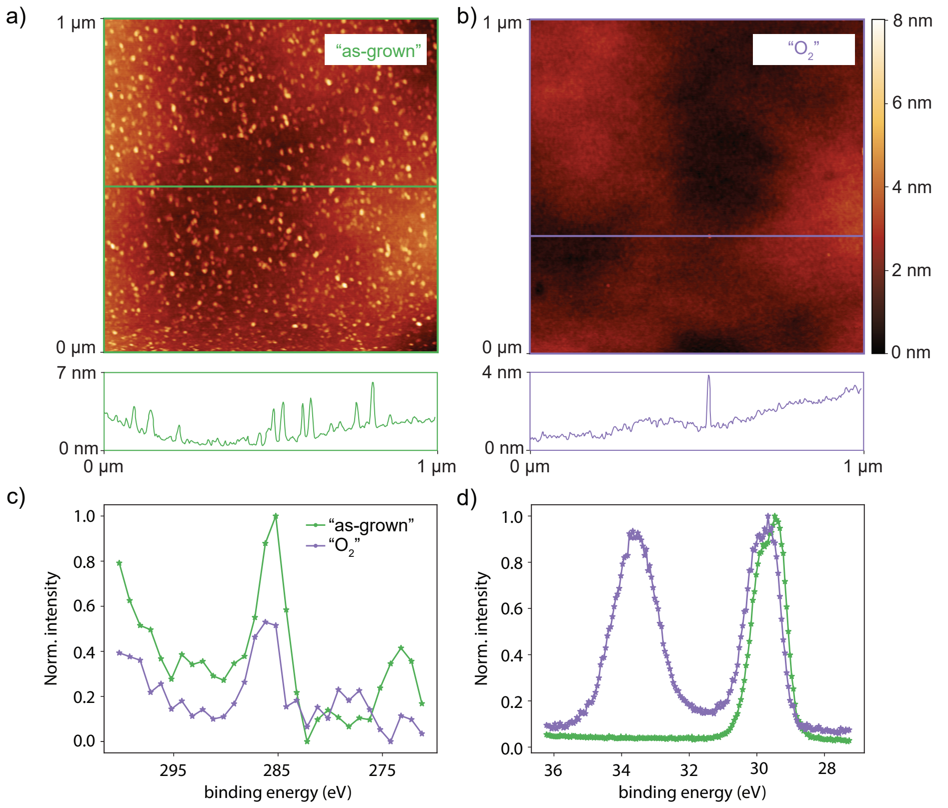

Fig. S5: X-ray photoelectron spectroscopy (XPS) and atomic force microscopy (AFM) data: Panels (a) and (b) contain AFM images of an "as-grown" and "O_2" sample, respectively. The line cuts display the larger modulation with the smaller spikes on top. Panels (c)-(d) show the normalized XPS detection intensity plotted as a function of binding energy around the carbon peak in (c) and around the GeO_2 and Ge3d peak in (d), respectively. The different colors correspond to measurements taken for the "as-grown" (green) and "O_2" (violet) samples.

the AFM tool, indicating that no damage has occurred during the oxygen plasma treatment. We also show zoomed-in images of areas $1\,\mu m^2$ in Figure S5(a)-(b) for the "as-grown" and "O_2" samples, respectively. Superimposed on the modulation, larger spikes are now visible for the "as-grown" sample. They almost completely disappear after oxygen plasma treatment. Judging from the XPS measurements (see below), these peaks probably are carbon contaminants.

The results of the XPS measurements are shown in Figure S5(c)-(d). The carbon content of the surface-near layers is reduced from 5.72 % in the "as-grown" sample to 2.04 % in the O_2 treated sample. The carbon likely originates from residual polymers from the photoresist

used for protecting the sample during dicing. Some of these polymer residuals have been removed by the oxygen plasma. However, all samples have been exposed to an additional lithography step (namely contact definition) after the O_2 treatment and before contact annealing, likely leading to new contamination. This indicates that the presence of carbon alone does not explain our findings but additional traps (e.g. from partially unoxidized silicon (Si)) exist. Furthermore, the O_2 plasma treated sample has a signature peak around 33 eV, indicating the presence of GeO_2. We conclude that the oxygen reached the silicon-germanium (SiGe) underneath the Si cap, and hence that the O_2 treatment was efficient in oxidizing the surface. Meanwhile, the absence of such a peak in the "as-grown" sample is in line with our hypothesis that the in-chamber oxidization of the Si cap after the growth was incomplete.

No XPS or AFM measurements were taken for the samples treated with HF. As HF does not efficiently passivate the Ge-rich SiGe underneath the Si cap, the exposure of the sample to atmosphere (which is necessary to perform these measurements) would lead to additional surface contamination. This prevents a comparative analysis.

Simulations of impurity-induced band-bending

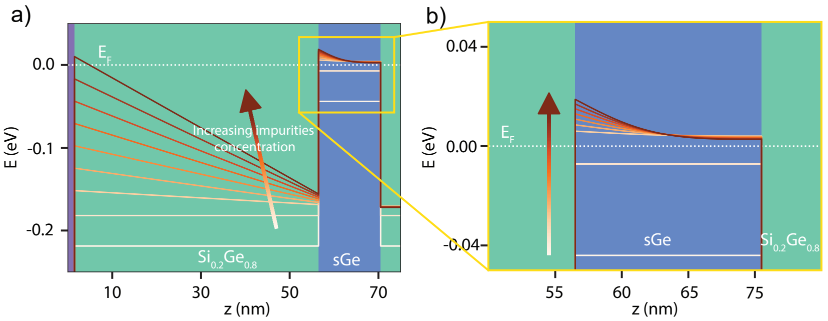

Fig. S6: Energy band simulations: (a) Heavy hole band energy diagram across a cross-section of the relevant part of the heterostructure including the QW. Different colors are used to indicate the increasing concentration of Boron impurities in the Si cap (purple region around $z = 0$) used for the simulations. A zoom into the strained germanium (sGe) QW region is provided in panel (b).

We use "NextNano", a selfconsistent multi-band k.p Schrödinger-Poisson-Current solver tool, to simulate the effects of impurities on the band structure. The impurities in the surface-near layers, e.g. the (partially oxidized) Si cap or the gate oxide, are simulated by adding a layer of boron-doped silicon on top of the heterostructure. Even though the exact doping concentration is probably not representative of the amount of traps, this allows us to qualitatively compare band alignments as a function of impurity concentration. In Figure S6, we plot the valence band energy at zero applied gate voltage for different doping concentrations. For low concentrations, the valence band stays below the Fermi energy accross the QW region, indicating highly resistive behaviour. Increasing the doping concentration, the valence band first crosses the Fermi energy in the QW region—indicating the accumulation of a hole gas without gate voltage—and then even crosses the Fermi level at the interface.

References

(1) Williams, K.; Gupta, K.; Wasilik, M. *Journal of Microelectromechanical Systems* **2003**, *12*, 761–778.

(2) Massai, L.; Hetényi, B.; Mergenthaler, M.; Schupp, F. J.; Sommer, L.; Paredes, S.; Bedell, S. W.; Harvey-Collard, P.; Salis, G.; Fuhrer, A.; Hendrickx, N. W. *Communications Materials* **2024**, *5*, 1–10.

(3) Cui, X.; Lee, G.-H.; Kim, Y. D.; Arefe, G.; Huang, P. Y.; Lee, C.-H.; Chenet, D. A.; Zhang, X.; Wang, L.; Ye, F.; Pizzocchero, F.; Jessen, B. S.; Watanabe, K.; Taniguchi, T.; Muller, D. A.; Low, T.; Kim, P.; Hone, J. *Nature Nanotechnology* **2015**, *10*, 534–540.

(4) Monroe, D.; Xie, Y. H.; Fitzgerald, E. A.; Silverman, P. J.; Watson, G. P. *Journal of Vacuum Science & Technology B: Microelectronics and Nanometer Structures Processing, Measurement, and Phenomena* **1993**, *11*, 1731–1737.

(5) Nigro, A.; Jutzi, E.; Forrer, N.; Hofmann, A.; Gadea, G.; Zardo, I. *Physical Review Materials* **2024**, *8*, 066201.

(6) Tracy, L. A.; Hwang, E. H.; Eng, K.; Ten Eyck, G. A.; Nordberg, E. P.; Childs, K.; Carroll, M. S.; Lilly, M. P.; Das Sarma, S. *Physical Review B* **2009**, *79*, 235307.
